# USP4 positively regulates RLR-induced NF-κB activation by targeting TRAF6 for K48-linked deubiquitination and inhibits enterovirus 71 replication

**DOI:** 10.1038/s41598-018-31734-6

**Published:** 2018-09-07

**Authors:** Chao Xu, Yang Peng, Qin Zhang, Xiao-Peng Xu, Xiang-Min Kong, Wei-Feng Shi

**Affiliations:** Department of Laboratory Medicine, the Third Affiliated Hospital of Soochow University, Changzhou, Jiangsu 213003 P. R. China

**Keywords:** Viral immune evasion, Genetics research

## Abstract

Retinoic acid-inducible gene I-like receptor (RLR) is one of the most important pattern recognition receptors of the innate immune system that detects positive and/or negative stranded RNA viruses. Subsequently, it stimulates downstream transcription of interferon regulatory factor 3 (IRF3) and nuclear factor κB (NF-κB) inducing the production of interferons (IFNs) and inflammatory cytokines. Tumour necrosis factor receptor associated factor 6 (TRAF6) is a key protein involved in the RLR-mediated antiviral signalling pathway, recruiting additional proteins to form a multiprotein complex capable of activating the NF-κB inflammatory pathway. Despite TRAF6 playing an important role in regulating host immunity and viral infection, the deubiquitination of TRAF6 induced by viral infection remains elusive. In this study, we found that enterovirus 71 (EV71) infection attenuated the expression of Ubiquitin-specific protease 4 (USP4) *in vitro* and *in vivo*, while overexpression of USP4 significantly suppressed EV71 replication. Furthermore, it was found that EV71 infection reduced the RLR signalling pathway and enhanced the degradation of TRAF6. USP4 was also found to interact with TRAF6 and positively regulate the RLR-induced NF-κB signalling pathway, inhibiting the replication of EV71. Therefore, as a novel positive regulator of TRAF6, USP4 plays an essential role in EV71 infection by deubiquitinating K48-linked ubiquitin chains.

## Introduction

EV71 is a single-stranded positive-sense RNA virus of the Picornaviridae family with a relatively large genome of approximately 7.5 kb^1^. Its single open reading frame encodes a polyprotein including three regions termed P1, P2, and P3. When EV71 infects cells, the P1 precursor is cleaved into four structural (VP1, VP2, VP3, and VP4) and seven non-structural proteins (2A, 2B, 2C, 3A, 3B, 3C, and 3D) by a protease^2,3^. Specifically, VP1 plays a central role in particle assembly and cell entry and is used in viral identification and evolution analysis^4^. It is well known that EV71 is a neurotropic picornavirus and often causes hand, foot and mouth disease. In addition it has been established that EV71 infects the human central nervous system, inducing fatal neurological manifestations such as brainstem encephalitis and cardiopulmonary complications^5^. EV71 is known to circulate endemically each year from summer through till fall, often leading to high morbidity with significant casualties among children^6^. In spite of multiple efforts, specific antiviral therapy is still not available for people who are infected with EV71.

As the first line of host defence, the innate immune system resists virus invasion predominantly by the sensing viral DNA and/or RNA by pattern recognition receptors (PRRs), which initiate signalling pathways ultimately leading to the secretion of inflammatory cytokines. It has been reported that toll-like receptors (TLRs) and retinoic acid-inducible gene I-like receptors (RLRs) are associated with the detection of viral infection by the host. Specifically, the RLRs consists of retinoic acid-inducible gene I (RIG-I), melanoma differentiation-associated protein 5 (MDA5), and a probable ATP-dependent RNA helicase DHX58 also known as RIG-I-like receptor 3 (LGP2)^7,8^. RIG-I/MDA5 sense viral RNA, and then interact with the adaptor protein mitochondrial antiviral-signalling protein (MAVS). Subsequently, the activation of MAVS recruits TRAF3 or TRAF6, facilitating the phosphorylation of IRF3 or NF-κB. This then enhances the expression of cytokines while inhibiting the replication of EV71, Sendai virus, and vesicular stomatitis virus^9^. Furthermore, the activation of NF-κB also induces the production of pro-IL-1β, NOD-like receptor family and pyrin containing 3 (NLRP3), and apoptosis associated speck-like protein containing CARD (ASC), resulting in the activation of caspase-1 and the maturation of IL-1β. The NF-κB pathway acts as an important mediator of inflammation, promoting the recruitment and activation of immune cells, as well as the release of secondary pro-inflammatory cytokines^10^. As previously reported, EV71 infection may induce the production of proinflammatory cytokines such as IL-1β, IL-6, and TNF-α; important in the development of disease^11,12^. For example, the concentrations of IL-1β and granulocyte colony-stimulating factor (G-CSF) in sera are significantly upregulated in EV71-infected patients that are cardiorespiratory compromised^13^.

Ubiquitination is an important post-translational modification in cells, maintaining homeostasis by regulating numerous cellular processes such as cell growth, proliferation, apoptosis, DNA damage response, and the immune response^14^. Within the cells, polyubiquitination plays several different roles depending upon the attachment position on the target proteins. Ubiquitin is a well conserved protein composed of 76 amino acids. There are seven lysine residues (K6, K11, K27, K29, K33, K48, and K63)^15^ in ubiquitin, and specifically from K48, the linked polyubiquitin chains regulate the proteasomal degradation of target proteins. Whereas, K63-linked and linear polyubiquitin chains control protein kinase activation and cell signalling. All the other ubiquitin’s are attached as monoubiquitin or polyubiquitin chains^16^.

Deubiquitination is the reversible reaction of ubiquitination, requiring deubiquitinating enzymes (DUBs). DUBs can induce the reversal of ubiquitin conjugation from target proteins, and ultimately lead to the regulation of downstream signalling^17^. The importance of many DUBs has been verified in the receptor tyrosine kinase signalling, signal transduction, gene transcription, DNA repair, proliferation, and mitosis^18–21^. Previously studies have shown that DUBs can regulate antiviral innate immune responses, such as A20, CYLD, USP21, and USP15, which function as negative regulators of NF-κB^22–26^. However, only one DUB, TRE17/USP6, has been shown to induce NF-κB activation^27^. Another DUB, USP4, has been confirmed to positively regulate RIG-I by removing K48-linked ubiquitin chains and participate in the cellular antiviral response^28^. Furthermore, USP4 can also positively regulate the function of IRF8 via K48-linked deubiquitination in regulatory T cells^29^.

In this study, we initially screened the differential expression of the USP family in EV71- infected human rhabdomyosarcoma (RD) cells, and found that the expression of USP4 was significantly downregulated, while NF-κB inflammatory signalling was inactivated. We also found that USP4 positively regulates RLR-induced NF-κB signalling while inhibiting EV71 replication, by targeting TRAF6 for K48-linked deubiquitination. As a novel positive regulator of RLR-induced NF-κB signalling, USP4 seemingly plays an essential role in the antiviral immune response.

## Results

### The expression of USP4 is inhibited upon EV71 infection *in vitro* and *in vivo*

**Figure 1:**
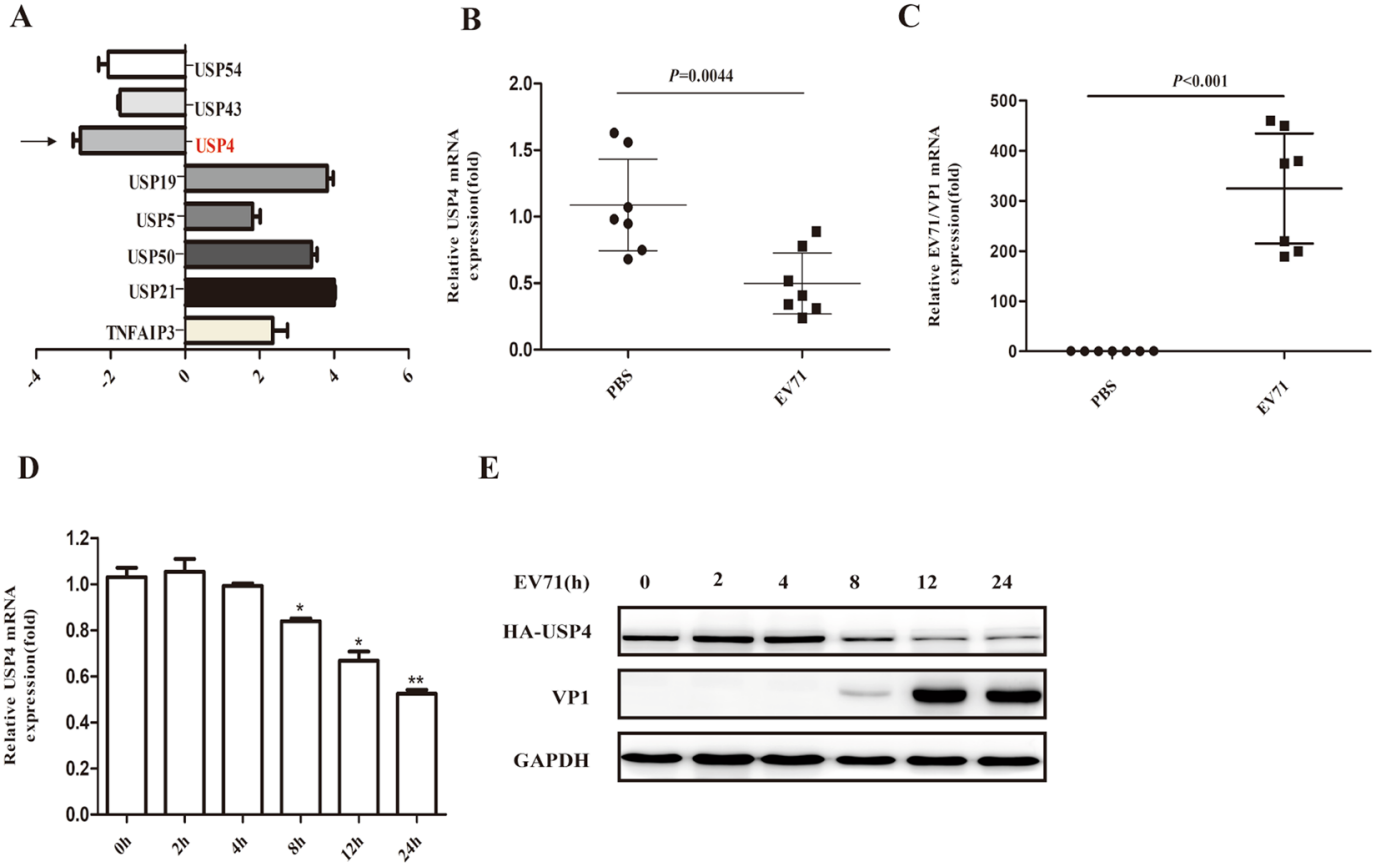
The expression of USP4 is inhibited upon EV71 infection *in vitro* and *in vivo*. (**A**) PCR array analysis of different gene expression levels of human DUBs in RD cells infected with EV71 for 8 h. (**B**) q-PCR analysis of the EV71/VP1 gene expression levels in the left-hind limb muscle tissue from EV71-infected AG129 mice. (**C**) q-PCR analysis of USP4 gene expression levels in the left-hind limb muscle tissue from EV71-infected AG129 mice. (**D**) The USP4 expression in EV71-infected RD cells (MOI = 0.5) was analysed by q-PCR at different time points. (**E**) The expression of USP4 and EV71/VP1 in EV71-infected RD cells (MOI = 0.5) was analysed by western blot at different time points. The EV71/VP1 protein expression was used as a control for viral infection. Data in (**A**–**D**) are shown relative to GAPDH expression and are presented as the mean ± SD, from three independent experiments. USP4 and VP1 are shown relative to GAPDH expression and are presented as the mean ± SD.*P < 0.05, **P < 0.01, and ***P < 0.001. The independent experiments were performed in triplicate.

To identify the potential DUBs that may be involved in EV71 infection, 88 common DUBs in EV71-infected RD cells were screened by PCR array. It was found that the expression levels of USP4, USP43, and USP54 were distinctly downregulated, while the levels of USP5, USP19, USP21, USP50, and TNFAIP3 were upregulated (Fig. 1A). Since USP4 had been previously suggested to function in important roles in the antiviral response^28^, we next focused on USP4 to study its regulatory roles in the antiviral immune response to EV71 infection. As EV71 can replicate efficiently in mice muscle tissue, a well-described mouse model for EV71 infection was established using AG129 mice lacking the IFN receptor. The mRNA levels of EV71/VP1 and USP4 were detected using quantitative – PCR (q-PCR) of the left-hind limb muscle tissues of EV71-infected AG129 mice. As shown in Fig. 1B,C, it was found that EV71/VP1 expression increased, while USP4 was consistently inhibited in EV71-infected AG129 mice. To investigate whether USP4 expression was affected by EV71 infection *in vitro*, RD cells were infected with EV71 for various lengths of time. Western blot and q-PCR analysis confirmed that the expression of USP4 was significantly downregulated 8 h, 12 h, and 24 h post-infection (p.i) (Fig. 1D,E). These results suggest that EV71 infection inhibit the expression of USP4 *in vitro* and *in vivo*.

### USP4 participates in antiviral immunity against EV71 infection

**Figure 2:**
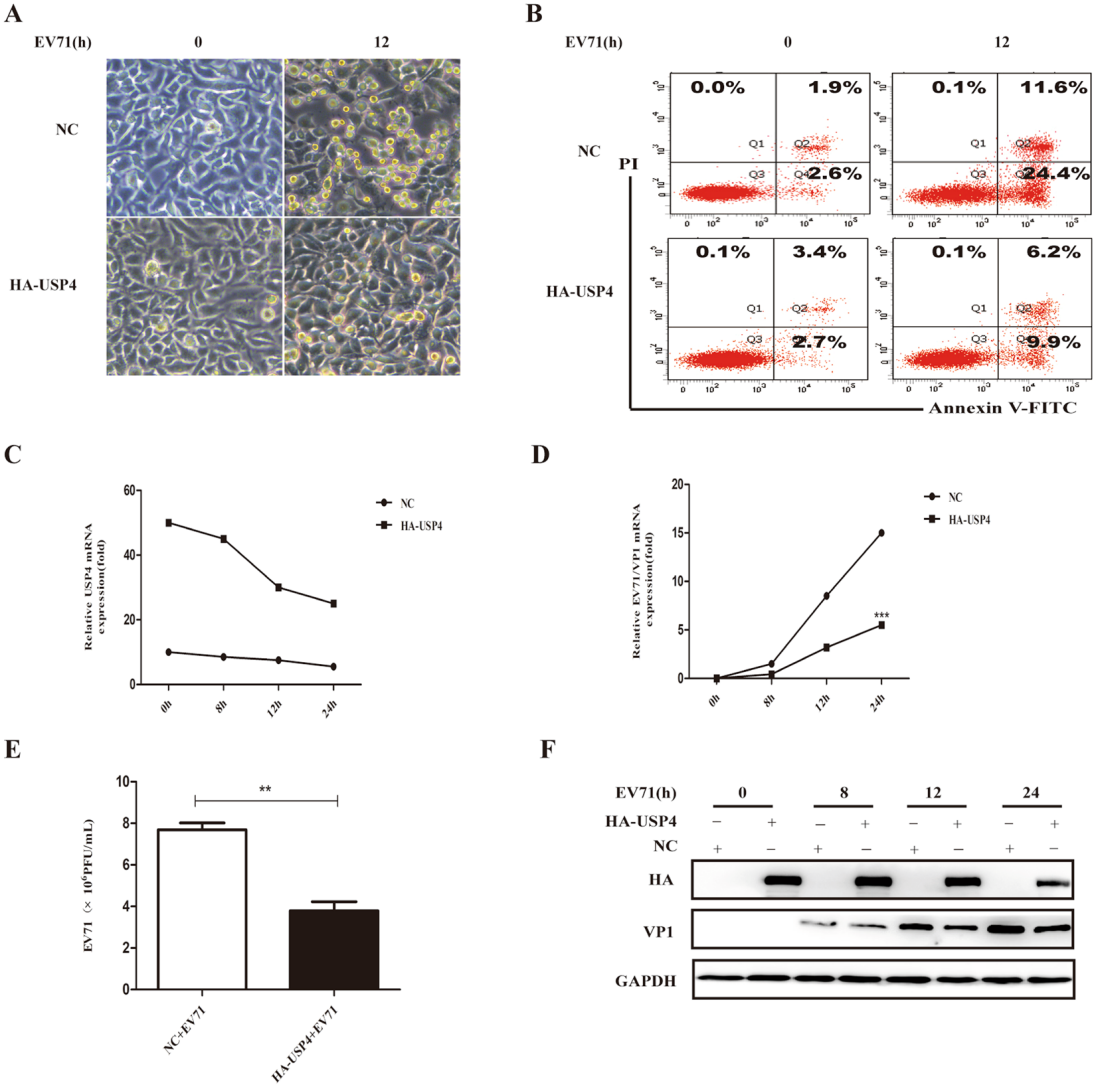
USP4 participates in antiviral immunity against EV71 infection. (**A**) Analysis of phase contrast microscopy of RD cells transfected with either an empty vector or USP4, and then infected with EV71 (MOI = 0.5) at the indicated time (magnification 20x). (**B**) Apoptosis assay of EV71-infected RD cells transfected with empty vector or USP4. The apoptosis assay was conducted using annexin V/FITC and PI double staining and flow cytometry. Q3, Q4, and Q2 represent normal cells, early apoptotic cells, and late apoptotic/necrotic cells, respectively. (**C**,**F**) q-PCR and western blot analysis of EV71 protein expression in RD cells transfected with control vector or HA-USP4 plasmids, followed by treatment with EV71 for variable lengths of time. (**E**) Viral titre measurement of EV71 propagating in RD cells transiently transfected with control vector or HA-USP4 plasmids. Data in (**D**,**E**) are presented as the mean ± SD. *P < 0.05, **P < 0.01, and ***P < 0.001. The independent experiments were performed in triplicate.

Having shown that the expression of USP4 is inhibited by EV71 infection, it was next thought to establish a link between the regulatory roles of USP4 in the antiviral immune response to EV71 infection. It was found that over-expression of USP4 enhanced the ability of RD cells to fight against invading EV71, and also reduced cell apoptosis (Fig. 2A,B). Meanwhile, transfection of USP4 suppressed the replication of EV71 and attenuated the expression of mRNA and protein by EV71/VP1 (Fig. 2D,F). As the overexpression USP4 of RD cells infected EV71, PCR assays also find that the expression levels of USP4 substantially reduced (Fig. 2C). Furthermore, the viral titre of EV71 in host cells was significantly decreased by the overexpression of USP4. (Fig. 2E). We also demonstrated that overexpression of the USP54 plasmid in RD cells did not affect proliferation of EV71 and cell apoptosis (Fig. S2). These findings indicate that USP4 may take part in antiviral immune response against invading EV71.

### EV71 inhibits the RLR signalling pathway and promotes the degradation of TRAF6

**Figure 3:**
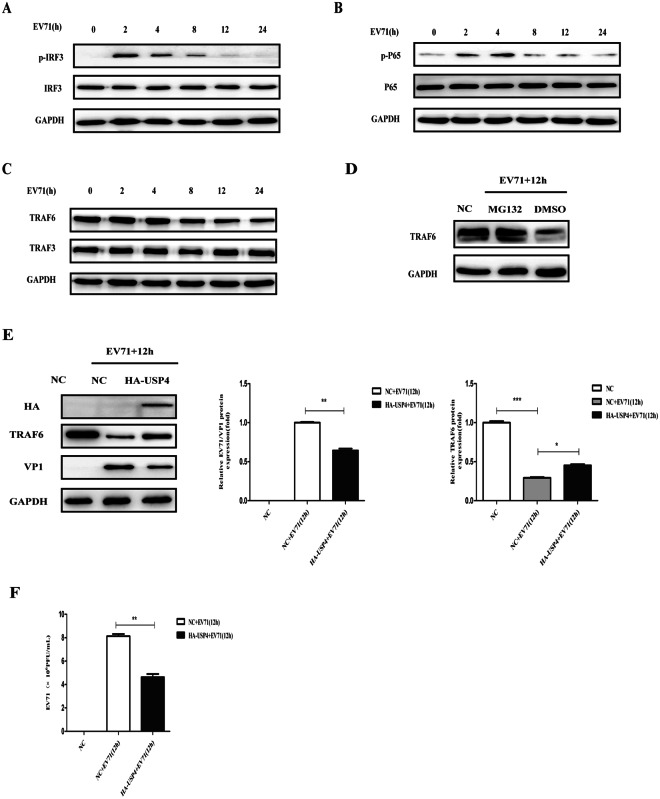
EV71 inhibits the RLR signalling pathway and promotes the degradation of TRAF6. (**A**) Western blot analysis of RD cells infected with EV71 for different durations of time. The cell lysates were analysed by immunoblotting using anti-pIRF3, anti-IRF3 and anti- GAPDH antibodies, respectively. (**B**) Western blot analysis of RD cells infected with EV71 for different duration of time. The cell lysates were analysed by immunoblotting using anti-pNF-κB P65, anti-NF-κB P65, and anti- GAPDH antibodies, respectively. (**C**) Western blot analysis of RD cells infected with EV71 for different durations of time. The cell lysates were analysed by immunoblotting using anti-TRAF6, anti-TRAF3, and anti- GAPDH antibodies, respectively. (**D**) To assess which pathway was involved in the degradation of TRAF6, cells were pre-treated with proteasome inhibitor MG132 at 5 μM for 12 h, followed by infection with EV71 (MOI = 0.5) for another 12 h. Cell lysates were prepared for western blot analyses with anti-TRAF6 antibodies. (**E**) Western blot analysis of RD cells transfected with control plasmid or HA-USP4 plasmid for 48 h, followed by treatment with EV71 (MOI = 0.5) for 12 h. The cell lysates were analysed by immunoblotting using anti-HA, anti-TRAF6, anti-VP1, and anti-GAPDH antibodies, respectively. (**F**) Viral titre of EV71 propagating in RD cells transiently transfected with control vector or HA-USP4 plasmids. Data in (**E**,**F**) are presented as the mean ± SD. *P < 0.05, **P < 0.01, and ***P < 0.001. The independent experiments were performed in triplicate.

RLR signalling is known to be crucial in the defence against viral infections. To investigate whether or not EV71 infection evokes RLR signalling, we infected RD cells with EV71 for various lengths of time. It was found that IRF3 and NF-κB P65 were activated at 2 h and 4 h p.i; however, the activation of IRF3 and NF-κB P65 gradually reduced as the infection continued (Fig. 3A,B). TRAF6 and TRAF3 are the crucial adaptor molecules for RLR-mediated NF-κB P65 and IRF3 activation, respectively. Western blot analysis showed that EV71 infection decreased the levels of TRAF6 in RD cells, but did not affect the expression of TRAF3 (Fig. 3C). Proteins are generally degraded by ubiquitin–proteasome dependent degradation. Pre-treated with MG132, an inhibitor of the proteasome, the degradation of TRAF6 was found to be blocked in a process induced by EV71 infection (Fig. 3D). However, when the RD cells were transfected with a plasmid bearing USP4, the results show that USP4 could decrease the degradation of TRAF6 and also inhibit the replication of EV71 (Fig. 3E). Furthermore, viral titre of EV71 in host cells was also significantly decreased with overexpression of USP4 (Fig. 3F). Therefore, as an upstream molecule, TRAF6 exhibits an essential role in the RLR-mediated signalling pathway.

#### USP4 positively regulates the antiviral NF-κB pathway

**Figure 4:**
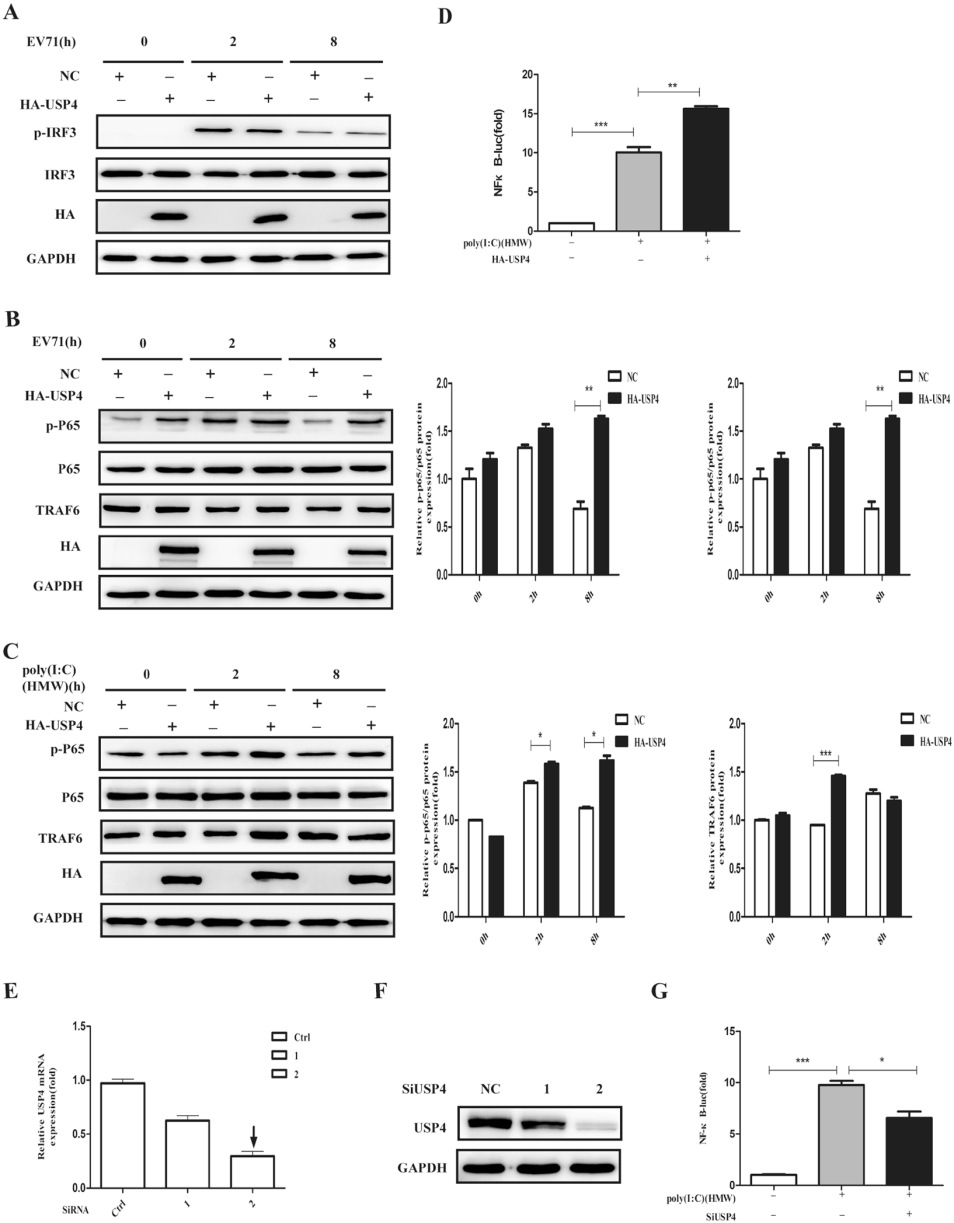
USP4 positively regulates the antiviral NF-κB pathway. (**A**) Western blot analysis of RD cells transiently transfected with control plasmid or HA-USP4 plasmid for 48 h, followed by treatment with EV71 for the indicated time. The cell lysates were analysed by immunoblotting using anti-pIRF3, anti-IRF3, anti-HA, and anti-GAPDH antibodies, respectively. (**B**) Western blot analysis of RD cells transiently transfected with control plasmid or HA-USP4 plasmid for 48 h, followed by treatment with EV71 for the indicated time. The cell lysates were analysed by immunoblotting using anti-pNF-κB P65, anti-NF-κB P65, anti-TRAF6, anti-HA, and anti- GAPDH antibodies, respectively. (**C**) Western blot analysis of HEK293T cells transfected with control plasmid or HA-USP4 plasmid for 48 h, followed by treatment with poly (I:C) (HMW; 30 μg/ml) for various lengths of time. The cell lysates were analysed by immunoblotting using anti-pNF-κB P65, anti-NF-κB P65, anti-TRAF6, anti-HA, and anti- GAPDH antibodies, respectively. (**D**) Luciferase activity in HEK293T cells transfected with a luciferase reporter for NF-κB, together with either a control plasmid or HA-USP4 expression plasmid, followed by either no treatment or treatment with intracellular HMW poly (I:C) (1 μg/ml). (**E**,**F**) q-PCR and western blot analysis of HEK293T cells transfected with control siRNA (Ctrl siRNA) or USP4-specific siRNA 1, siRNA 2 for 48 h. (**G**) Luciferase activity in HEK293T cells transfected with a luciferase reporter for NF-κB, together with either control siRNA or USP4-specific siRNA 2, followed by either no treatment or treatment with intracellular HMW poly (I:C) (1 μg/ml). Data in (**B**–**D**,**G**) are presented as the mean ± SD. *P < 0.05, **P < 0.01, and ***P < 0.001. The independent experiments were performed in triplicate.

Next, we investigated whether USP4 had an effect on NF-κB or the type I IFN signalling pathway. Transfection of USP4 resulted in the phosphorylation of NF-κB P65 and the levels of TRAF6 in RD cells increased (Fig. 4B). In contrast, USP4 expression had no obvious effect on IRF3 (Fig. 4A). Previous studies have shown that EV71 could trigger antiviral IFN-I signalling and the NF-κB pathway through the accessary protein MDA5^30^, and furthermore poly(I:C) of high molecular weight (HMW) has also been demonstrated as a ligand for MDA5-mediated type I IFN signalling and NF-κB pathway activation. It was found that transfection of USP4 consistently increased the sensitivity of HEK293T cells to HMW Poly(I:C)-induced NF-κB P65 activation (Fig. 4C), consistent with the results observed in RD cells. In order to further determine whether USP4 plays a crucial role in NF-κB activation induced by HMW Poly(I:C), USP4 expression plasmids were transfected into HEK293T cells, and subsequently treated with intracellular HMW poly(I:C). It was found that USP4 significantly promoted the activities of NF-κB luciferase reporter gene (Fig. 4D). Therefore, to further confirm USP4-induced NF-κB activation, we designed and synthesized two siRNAs to knock down the intrinsic expression of USP4. When the two siRNAs were compared it was found that siRNA2 downregulated the mRNA and protein expression of USP4 with a higher efficiency, and was therefore used in the following experiments (Fig. 4E,F). Remarkably, knockdown of USP4 inhibited the activities of the NF-κB luciferase reporter gene that were induced by HMW poly(I:C) (Fig. 4G). Taken together, USP4 positively regulates the activation of NF-κB in an EV71 infection.

### USP4 targets TRAF6 through its USP domain

**Figure 5:**
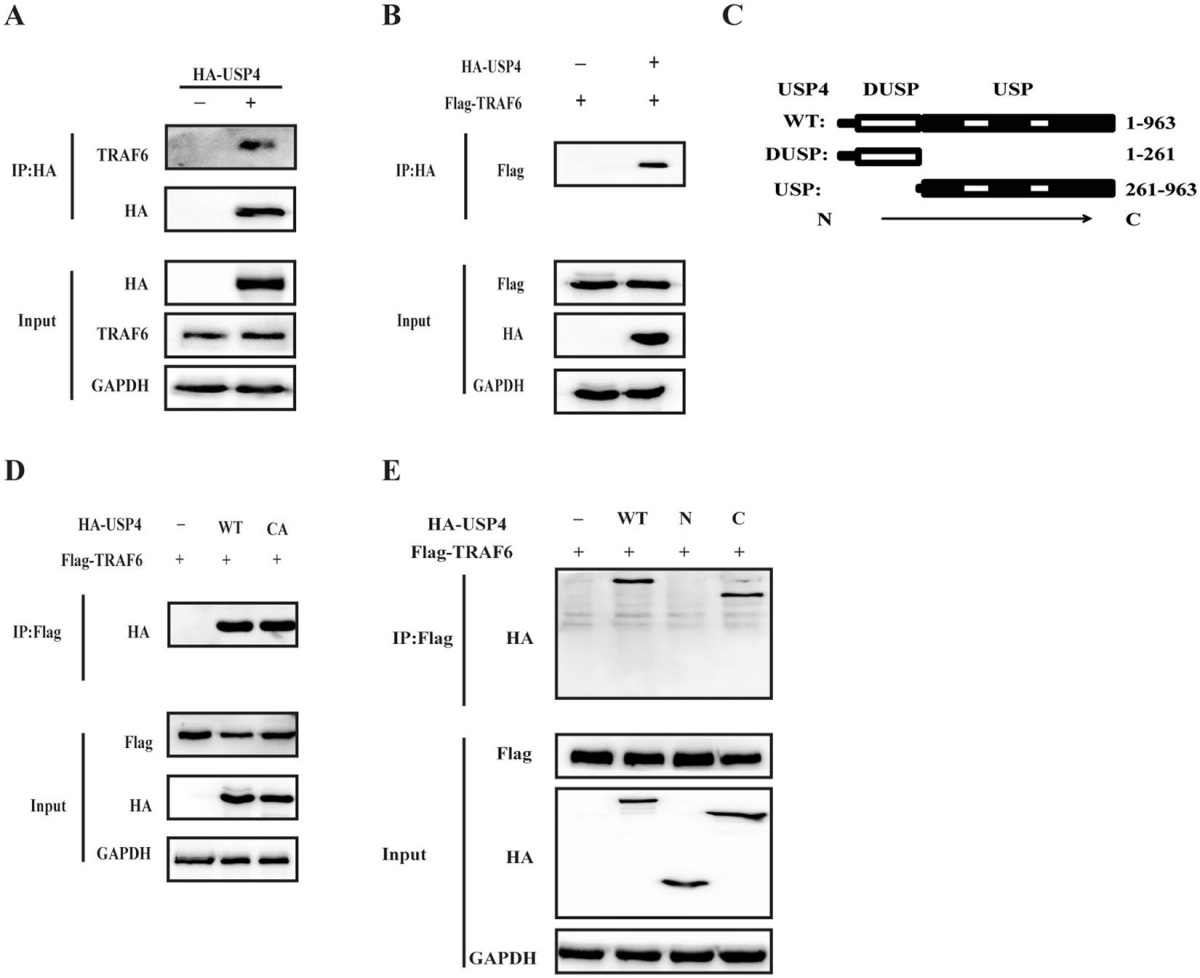
USP4 targets TRAF6 through its USP domain. (**A**) HEK293T cells transfected with either HA-USP4 or a control vector for 48 h were lysed and immunoprecipitated with anti-HA agarose beads. This was followed by western blot analysis of eluted immunocomplexes with TRAF6. (**B**,**D**) HEK293T cells transfected with Flag-TRAF6, together with either HA-USP4, mutated HA-USP4 (CA) or an empty vector were collected 48 h post-transfection and immunoprecipitated with either anti-HA agarose beads (**B**) or anti-Flag agarose beads (**D**). (**C**) USP4 wild-type (WT) contains a DUSP domain and a C-terminal USP domain. N260 lacks the C-terminal USP domain, C261 lacks the DUSP domain. (**E**) Lysates from HEK293T cells co-transfected with Flag-TRAF6 and either full-length HA-USP4 (WT) or its truncated constructs (N or C), and then were subjected to immunoprecipitation with anti-Flag agarose beads followed by SDS-PAGE analysis with anti-HA-HRP antibody). The independent experiments were performed in triplicate. Please note the mutated HA-USP4 (CA) expression construct was generated using site-directed mutagenesis(G-C).

To fully understand the function of USP4 in the NF-κB pathway, further study was performed to identify which molecular target USP4 targets. As previously reported, TRAF6 is a key adaptor molecule for the activation of the NF-κB pathway^31^, therefore it was speculated that USP4 indeed interacts with TRAF6. HA-tagged USP4 plasmids were transfected into HEK293T cells, and it was found that TRAF6 was specifically pulled down by USP4 by intrinsic immunoprecipitation (IP) (Fig. 5A). Flag-TRAF6 and HA-USP4 plasmids were co-transfected into HEK293T cells and after 48 h, further IP experiments were performed with anti-HA antibody, and it was established that USP4 interacted with TRAF6 (Fig. 5B). USP4 contains an ubiquitin-specific domain at its C-terminal end and cys-311 is known to be required for the deubiquitinase activity of USP4 (Fig. 5C). As shown in Fig. 5D, the interaction between USP4 and TRAF6 was not impaired by the mutation of the Cys-311 to Alanine (‘CA mutant’) of USP4 molecule. To further map the functional domain that interacts with TRAF6, we generated two truncated constructs based on the structural domains of USP4 and as shown in Fig. 5E, only the C-terminal domain (C) of USP4 was able to interact with TRAF6.

### USP4 removes K48-linked polyubiquitination from TRAF6

**Figure 6:**
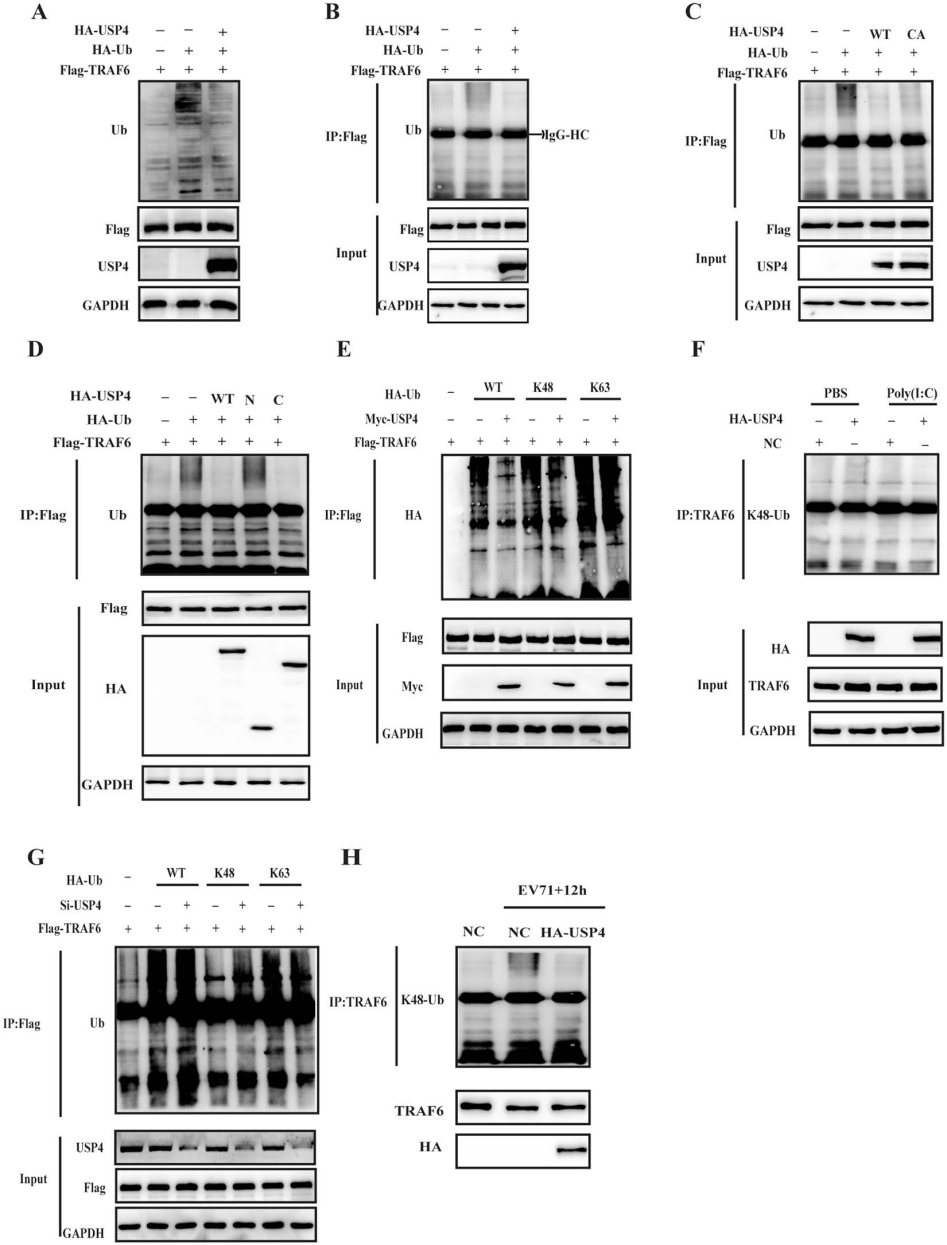
USP4 removes K48-linked polyubiquitination from TRAF6. (**A**) Western blot analysis of HEK293T cells transfected with Flag-TRAF6, HA-USP4, and HA-Ub for ubiquitination levels; GAPDH was used as a loading control. (**B**,**C**) HEK293T cell lysates transfected with Flag-TRAF6, HA-Ub, together with either wild-type HA-USP4 (WT) or mutated HA-USP4 (CA) were collected and immunoprecipitated with anti-Flag agarose beads. The eluted immunocomplexes was then subjected to SDS-PAGE analysis with anti-ubiquitin antibody. (**D**) Lysates from HEK293T cells transiently expressing Flag-tagged TRAF6 and HA-tagged ubiquitin, together with either HA-tagged USP4 or its truncated constructs (N or C) were immunoprecipitated with anti-Flag agarose beads; the eluted protein complexes was subjected to western blot analysis with anti-ubiquitin antibody. (**E**) HEK293T cells transfected with Flag-TRAF6, Myc-USP4, together with either WT HA-Ub or its mutants (K48 or K63) were harvested and subjected to immunoprecipitation with anti-Flag agarose beads followed by SDS-PAGE analysis with HA antibody. (**F**) Lysates from HEK293T cells transfected with HA-USP4 or a control vector followed by treatment with poly (I:C) (HMW; 30 μg/ml) for 8 h were subjected to immunoprecipitation with an anti-TRAF6 antibody. This was followed by western blot analysis of eluted immunocomplexes with K48 linkage-specific ubiquitin antibodies. (**G**) HEK293T cells transfected with Flag-TRAF6, control siRNA or USP4-specific siRNA, together with either WT HA-Ub or its mutants (K48 or K63) were harvested and subjected to immunoprecipitation with anti-Flag agarose beads followed by SDS-PAGE analysis with anti-ubiquitin antibody. (**H**) Western blot analysis of RD cells transfected with control plasmid or HA-USP4 plasmid for 48 h, followed by treatment with EV71 (MOI = 0.5) for 12 h. The cell lysates were subjected to immunoprecipitation with anti-TRAF6 antibody, followed by western blot analysis of eluted immunocomplexes with K48 linkage-specific ubiquitin antibodies.

Having shown that USP4 targets and interacts with TRAF6, we further studied the cellular functions of USP4 toward TRAF6 ubiquitination. Flag-TRAF6, HA-USP4, and HA-Ub plasmids were co-transfected into HEK293T cells and it was found that USP4 notably decreased overall ubiquitination levels (Fig. 6A). To determine whether or not USP4 specifically has the deubiquitinating effect on TRAF6, flag-tagged TRAF6 was pull down and examined the deubiquitinating ability of USP4 on TRAF6. From this it was found that USP4 exhibited the deubiquitinating effect on TRAF6 (Fig. 6B). Furthermore, the deubiquitinating effect of USP4 on TRAF6 was not dramatically impaired by the mutation on the enzymatic active site of USP4 at C311A (Fig. 6C). These findings suggest that USP4 deubiquitinates TRAF6 largely in a noncatalytic manner. Subsequently, we detected if any particular domain of USP4 is essential for the deubiquitination of TRAF6. As shown in Fig. 6D, the ubiquitin-specific domain of USP4 was sufficient to deubiquitinate TRAF6, while the N-terminal domain had no deubiquitinating effect, compared with full-length USP4. Taken together, these findings suggest that USP4 decreases the polyubiquitination of TRAF6 in a ubiquitin-specific domain-dependent manner. HEK293T cells were then transfected with WT ubiquitin plasmid or Lys-mutated ubiquitin plasmids (K48/K63), and it was found that TRAF6 was modified identically through the K48-linked polyubiquitin chains and this type of modification could be reversed by USP4. However, we did not observe any apparent ubiquitination of TRAF6 by K63-linked polyubiquitin chains (Fig. 6E). Following on from this, the USP4-knockdown mediated deubiquitination of TRAF6 was further explored and as shown in Fig. 6G, K48-linked polyubiquitin chains were enhanced significantly in the USP4-knockdown cells. Finally, the endogenous ubiquitination levels of TRAF6 were explored following USP4 overexpression, and it was observed that the extent of K48-linkage was inhibited significantly in these cells; an essential factor in the activation of the NF-κB pathway (Fig. 6F). As shown in Fig. 6H, it was found that EV71 infection induced K48-linked polyubiquitination of TRAF6, and HA-USP4 decreased K48-linked polyubiquitination of TRAF6 as well as the stability of TRAF6 in EV71 infected cells. These data indicate that USP4 positively regulates the RLR-mediated activation of NF-κB by removing the K48-linked polyubiquitin chains from TRAF6.

## Discussion

The process of reversing ubiquitination is performed by a family called DUBs. Currently, more than 100 DUBs have been identified in the human genome^32^. DUBs can be divided into sub families based upon their enzyme features and these include a metalloprotease family and a cysteine protease family, with the latter further divided into four subclasses, including USPs (ubiquitin-specific proteases), ubiquitin C-terminal hydrolases (UCHs), otubain proteases (OTUs), and Machado–Joseph disease proteases (MJDs). However, only a minority of them have been functionally characterized. The USPs are the biggest subfamily with more than 50 members^33^, with some of them known to regulate antiviral immune responses^28,34,35^. Previous studies have indicated that EV71 infection induces the up-regulation of USP19, which negatively regulates the antiviral type I interferon signalling by removing K-63 polyubiquitin chains from TRAF3^35^. In this study, 88 DUBs were screened in EV71-infected RD cells, and the expression of USP4 was found to be remarkably decreased at 8 h p.i. as the EV71/VP1 expression gradually increased. Consistent with the result *in vitro*, the expression of USP4 was significantly downregulated in EV71-infected AG129 mice *in vivo*. In order to confirm the relationship between USP4 and EV71 infection, USP4 was transfected into EV71-infected RD cells and it was found that the overexpression of USP4 decreased the mRNA and protein expression of EV71/VP1, inhibiting the replication of EV71, while also reducing RD cell apoptosis. This indicates that USP4 improved the ability of RD cells to defend EV71. Therefore, we speculate that USP4 plays an essential role in antiviral immune response. USP4 has previously been confirmed to target TRAF2 and TRAF6 for deubiquitination and it has been established that USP4 inhibits TNFα-induced cancer cell migration^35^. It was confirmed that USP4 associates with TRAF2 and TRAF6, consistent with our results. However, there is no evidence of how USP4 deubiquitinates TRAF6 and TRAF2 proteins; our results prove that USP4 targets TRAF6 for K48-linked deubiquitination. It was also found that USP4 inhibited NF-κB activation, but in this study we confirmed that USP4 positively regulates the NF-κB signalling pathway during EV71 infection. During viral infection (Sendai virus), USP4 has been found to remove K48-linked polyubiquitination chains from RIG-I and positively regulate the RIG-I-mediated antiviral response^28^. However, Sendai virus and EV71 are different types of viruses that can be recognized by different receptors^30^. Therefore, we found that overexpression of USP4 activates the phosphorylation of NF-κB p-P65, but it had no obvious effect on IRF3.

It is well-known that RLR induces the activation of both NF-κB through TRAFs/TAK1/IKKs and IRF3 through TBK1, leading to the production of proinflammatory cytokines and IFN-α, respectively^36^. Both TRAF2 and TRAF6 are key signalling proteins in most NF-κB signalling pathways, executing similar roles either independently or together in different NF-κB activation pathways. Many researchers have highlighted that TRAF6 plays a pivotal role in RLR-mediated signalling pathway^37–39^. Normally, TRAF6 forms a homodimer and catalyses K63-linked ubiquitination; however, recently it was found that TRAF6 can also be modified by K48-linked ubiquitination, promoting TRAF6 degradation through the proteasome, blocking the inflammatory signal. Therefore, this balance between the K48 and K63-linked ubiquitination may trigger a cascade response towards either anti- or pro-inflammatory response^40,41^. USP4 is generally reported as a cysteine protease that removes ubiquitin chains in several signalling pathways^42,43^. Furthermore, TRAF6 has been identified as a target of USP4 in TNF-α induced NF-κB activation^44^. In this study it was established that USP4 exhibits a critical role in positively regulating virus-induced NF-κB activation. Further study also confirmed that the USP domain of USP4 has the ability to interact with TRAF6, removing the K48-linked polyubiquitination and stabilized TRAF6, ultimately leading to an increasing production of proinflammatory cytokines.

The transcription factor NF-κB plays a number of crucial roles in immunity by regulating the expression of both inducers and effectors at various times throughout the expansive networks that define the responses to pathogens^45^. It is activated by distinct but partially shared pathways. PRRs, such as TLRs, RLRs, C-type lectin receptors (CLRs) and NOD-1 like receptors (NLRs) sense the invasion of pathogens and initiate signalling to activate NF-κB or IRF, resulting in the enhanced expression of proinflammatory cytokines such as IL-6, TNF-α, and certain IFNs^46^. All these cytokines regulate the inflammatory responses to ensure the invading pathogens are eliminated, and NF-κB is a key factor^47^. Type I IFN signalling and NF-κB pathway have been shown to be activated during the early stage of EV71 infection, while EV71 suppressed the activation of IRF3 and NF-κB P65 as the infection continued; similar to this study^48^. Previous study also showed that USP4 targets TAK1 by removing K63-linked polyubiquitination and then recruit IκB kinase (IKK) complex to simulate TNF-α -induced NF-κB activation^49^. However, we found that EV71 infection inhibit the expression of USP4 to attenuate the activation of the NF-κB pathway, an example of an evolved mechanism through which EV71 antagonizes host defences.

From this study it can be speculated that EV71 infection inhibits USP4 expression and promotes TRAF6 polyubiquitination of K48-linkage as a way to regulate the proteasomal degradation of TRAF6. Additionally, we confirmed that EV71 can multiply in host cells by suppressing the NF-κB signalling pathway. In summary, a previously unrecognized role of USP4 was identified in the positive regulation of RLR-induced NF-κB signalling and antiviral immunity. USP4 specifically targets ubiquitinated TRAF6 and then cleaves the polyubiquitin chains. Our findings provide novel insights into the molecular mechanisms by which USP4 positively regulates NF-κB signalling and thus plays a critical role in maintaining the balance between innate immune responses and immune tolerance.

## Methods

### Materials

Antibodies for USP4(2651) phosphorylated NF-κB p65(3033), NF-κB p65(8242), Myc(2272), and TRAF6 (8028) were purchased from Cell Signalling Technology (USA); horseradish peroxidase (HRP) conjugated goat anti-rabbit IgG secondary antibodies (L3012) were purchased from Signalway Antibody (USA); antibodies against EV71/VP1 (169442), K48 linkage-specific ubiquitin (140601), and horseradish peroxidase (HRP) conjugated goat anti-mouse IgG secondary antibodies (97023) were purchased from Abcam (UK); anti-GAPDH and anti-hemagglutinin (HA)-HRP (561–7) were purchased from Protein TECH Group (Chicago, USA) and MBL (Japan), respectively; antibodies against TRAF3 (SC-1828) and ubiquitin (SC-8017) were purchased from Santa Cruz Biotechnology (USA); proteasome inhibitors MG132 (S2619) were purchased from Selleck; anti-Flag (M2)-horseradish peroxidase (A8592) was purchased from Sigma (USA); Poly (I:C) (HMW) was purchased from InvivoGen (CA, USA).

### Cell culture

The rhabdomyosarcoma (RD) cells and HEK293T cells (China Centre for Type Culture collection) were cultured in Dulbecco modified Eagle medium (DMEM) (HyClone, USA) and supplemented with 10% foetal bovine serum (Gibco, USA) and 100 U/ml penicillin–streptomycin solution (Invitrogen, USA). Cells were cultured at 37 °C in a 5% CO_2_ atmosphere.

### Virus infection

The EV71 strain (BrCr strain, ATCC VR784, GenBank accession number: U22521) was provided by China Centre for Type Culture collection. For viral infections, RD cells were infected with EV71 at a multiplicity of infection (MOI) value of 0.5 in half-volume 2% serum-reduced medium to allow for viral adsorption for 1.5 h. This was followed by replacement with full-volume maintenance medium for different durations. Infection of EV71 for 0 h in the experiment represents cells that were mock-infected with an equal volume of phosphate-buffered saline (PBS). The expression of the viral protein VP1 was used as a control for each experiment.

### EV71 plaque assay and detection of virus replication

The RD cells were transfected with the plasmids or siRNA for 48 h prior to EV71 infection (MOI = 0.5). After 1.5 h infection, cells were washed with PBS three times and then fresh media was added. After which, the supernatants were harvested after 24 h. The supernatants were diluted 1:10^6^ and used to infect RD cells cultured in 96-well plates. At 1.5 h post-infection (p.i), the supernatant was removed and 3% methylcellulose was added. At 3 days p.i, the methylcellulose was removed, the cells were fixed with 4% formaldehyde for 20 min, and subsequently stained with 0.2% crystal violet. Plaques were counted, averaged, and multiplied by the dilution factor to determine viral titre as PFU/ml.

### RNA quantitation

Quantitative Real-time PCR (qRT-PCR) was performed using an Applied Biosystems 7500 Real-Time PCR System (USA). Total RNA was isolated using an RNA Purification Kit (TianGen, Beijing, China) and was reverse transcribed at 42 °C for 15 min using a PrimeScript RT Reagent kit (TaKaRa, Japan) to obtain cDNA. Then, the cDNA was diluted 40 times and amplified using SYBR Premix Ex Taq II PCR mix (TaKaRa, Japan). GAPDH was used as the internal control. The parameters for the PCR reaction were as follows: 94 °C for 10 min, followed by 40 cycles of 94 °C for 30 s, 54 °C for 30 s, and 72 °C for 30 s. The primers for real-time PCR are shown in Table 1. Finally, the CT values for each reaction were collected and the changes in the expression of the target gene were normalized to GAPDH, and calculated using the following formula: Relative mRNA level of target gene (folds of control) = 2^−ΔΔCT^.

**Table 1:** Primer pairs for real-time PCR.

Gene	Sequence
USP4	F: 5′-CCTGGGCTCTGTGGACTTG-3′
	R: 5′- TGTTGATTTCGGCTTCATACTC-3′
EV71/VP1	F: 5′-GAGTGGCAGATGTGATT GA-3
	R: 5′-TCCAGTGTCTAAGCGATGA-3′
GAPDH (human)	F: 5′-TATGACAACAGCCT CAAGA-3′
	R: 5′-ATGAGTCCTTCCACGATAC-3′
GAPDH (mouse)	F: 5′-AACCTGCCAAGTATGATGA-3′
	R: 5′-GGAGTTGCTGTTGAAGTC-3′

### Animal model for EV71 infection

All procedures involving experimental animals were performed in accordance with the Ethical Guidelines for Animal Care of the Soochow University Health Science Centre and the experimental protocols were approved by Ethics Committee of Soochow University Health Science Centre based on <Laboratory Animal-Requirements of Environment and Housing Facilities (GB 14925-2001)> and <Soochow Administration Rule of Laboratory Animal>. AG129 mice at 12 days of age were infected with 1 × 10^7^ plaque forming units of EV71 or mock-infected with an equal volume of PBS via intraperitoneal injection. Three days after infection, mice were sacrificed and analysed for USP4 and EV71/VP1 gene expression levels in the left-hind limb muscle tissue.

### Assay of luciferase activity

HEK293T cells were seeded in 6-well plates at 5 × 10^5^ cells per well and cultured overnight. The cells were transfected with the plasmids or SiRNA, together with a NF-κB dependent firefly luciferase construct, and a renilla luciferase construct, which was used to normalize luciferase activity. At 36 h post transfection, 1 μg/ml of poly (I:C) was added to the media. The cells were incubated for an additional 4 h before they were collected for dual luciferase reporter gene assays. Luciferase activity was detected using a Dual-Luciferase Reporter Assay System (Promega). The relative luciferase activity was calculated by dividing the firefly luciferase activity by the renilla luciferase activity.

### Cell apoptosis assay

RD cells were seeded at 3 × 10^5^ per well in 6-well plates and cultured overnight. The cells were then transfected with the plasmids for 24 h, followed by infection with EV71 at a MOI of 0.5. After 12 h, the cells were washed twice with cold PBS and harvested. Cell apoptosis was subsequently determined using an annexin V/PI-based apoptosis detection kit (BD Biosciences) and analysed using a flow cytometer (BD FACSCanto II).

### Sequences, plasmids & transfection

pIRES2-EGFP vector containing HA-USP4, HA-USP4-C311A, HA -USP, HA- DUSP, and Myc-USP4 were purchased from Transheep Bio (Shanghai, China). The Flag-TRAF6 plasmid, wild-type (WT) HA-tagged ubiquitin, and its mutated plasmids (K48: only Lysine (Lys, K)-48 retained; K63: only Lys-63 retained) were kindly gifted from Prof. Zheng (Soochow University, China). All the plasmids were confirmed by sequencing. For transient overexpression, plasmids were transfected into cells for 48 h using Lipofectamine 2000 (Invitrogen), according to the manufacturers protocol. Transient gene silencing with small-interfering RNA (siRNA) was performed using INTERFERin (Polyplus Transfection, Illkirch, France). Target sequences for silencing USP4 were synthesized by RiboBio (Guangzhou, China) and listed as 5′-GCΜGGGACAΜGUACAAΜGU-3′(siRNA1), 5′-GGCUCΜGGAACAAAUACAU-3′(siRNA2), and a scrambled control sequence of 5′-UUCUCCGA ACGΜGUCA CGU-3′.

### Immunoprecipitation assay

For immunoprecipitation (IP), HEK293T cells that were co-transfected with Flag-TRAF6 and HA-USP4 for 48 h were harvested and lysed on ice with a cell lysis buffer containing 20 mM Tris-HCl (pH 8.0), 150 mM NaCl, 1% (v/v) Triton X-100, 10% (v/v) glycerol, 0.5 mM DTT, 1 mM Na_3_VO_4_, and 25 mM β-glycerol-phosphate, together with 1 mM PMSF, a complete protease inhibitor cocktail (Roche, Basel, Switzerland), and a phosphatase inhibitor cocktail (Roche). Whole cell lysates were collected by centrifugation for 20 min at 12,000 × *g* and were incubated with a HA antibody or Flag antibody for 1 h at 4 °C. This was followed by an overnight incubation with protein A/G plus agarose beads (Santa Cruz) at 4 °C. After which the beads were collected via centrifugation for 1 min at 3000 × *g* and washed six-times with the lysis buffer. The immunoprecipitants were eluted from the beads and subjected to western blot analysis; 1–2% whole cell lysates (Input) served as a control for the IP.

### Deubiquitination assays

Deubiquitination assays were conducted as described previously^35^. In brief, for analysing the ubiquitination of TRAF6, HEK293T cells were co-transfected with either Flag-TRAF6, HA-tagged ubiquitin or its mutants, with or without HA-USP4 for 48 h. Whole cell lysates were incubated with a Flag antibody for 1 h at 4 °C, which was followed by incubation with Protein A/G plus agarose beads overnight at 4 °C. After which the beads were collected via centrifugation for 1 min at 3000 × *g* and washed six-times with lysis buffer. The immunoprecipitants were eluted from the beads and subjected to western blot analysis with anti-ubiquitin antibodies. Western blot analysis of whole cell lysates served as the control for the deubiquitination assay.

### Statistical analysis

Data were expressed as means ± standard deviation. The significance of differences was evaluated using one-way analysis of variance (ANOVA) or t-test implemented in the software package SPSS statistics v17.0. Difference were considered to be significant at *P* < 0.05.

## Electronic supplementary material

Supplementary Information

## References

[1] Solomon T (2010). Virology, epidemiology, pathogenesis, and control of enterovirus 71. The Lancet. Infectious diseases.

[2] Zhang Y (2018). Enterovirus 71 inhibits cytoplasmic stress granule formation during the late stage of infection. Virus research.

[3] McMinn PC (2002). An overview of the evolution of enterovirus 71 and its clinical and public health significance. FEMS microbiology reviews.

[4] Chang CK (2018). Mutations in VP1 and 5′-UTR affect enterovirus 71 virulence. Scientific reports.

[5] Ooi MH, Wong SC, Lewthwaite P, Cardosa MJ, Solomon T (2010). Clinical features, diagnosis, and management of enterovirus 71. The Lancet. Neurology.

[6] Yi L, Lu J, Kung HF, He ML (2011). The virology and developments toward control of human enterovirus 71. Critical reviews in microbiology.

[7] Loo YM (2008). Distinct RIG-I and MDA5 signaling by RNA viruses in innate immunity. Journal of virology.

[8] Takeuchi O, Akira S (2010). Pattern recognition receptors and inflammation. Cell.

[9] Loo YM, Gale M (2011). Immune signaling by RIG-I-like receptors. Immunity.

[10] Dinarello CA (2009). Immunological and inflammatory functions of the interleukin-1 family. Annual review of immunology.

[11] Gong X (2012). Excessive proinflammatory cytokine and chemokine responses of human monocyte-derived macrophages to enterovirus 71 infection. BMC infectious diseases.

[12] Wang W (2017). EV71 3D Protein Binds with NLRP3 and Enhances the Assembly of Inflammasome Complex. PLoS pathogens.

[13] Griffiths MJ (2012). In enterovirus 71 encephalitis with cardio-respiratory compromise, elevated interleukin 1beta, interleukin 1 receptor antagonist, and granulocyte colony-stimulating factor levels are markers of poor prognosis. The Journal of infectious diseases.

[14] Nath SR, Lieberman AP (2017). The Ubiquitination, Disaggregation and Proteasomal Degradation Machineries in Polyglutamine Disease. Frontiers in molecular neuroscience.

[15] Pickart CM (2001). Mechanisms underlying ubiquitination. Annual review of biochemistry.

[16] Malynn BA, Ma A (2010). Ubiquitin makes its mark on immune regulation. Immunity.

[17] Nijman SM (2005). A genomic and functional inventory of deubiquitinating enzymes. Cell.

[18] Vogel RI (2015). Simultaneous inhibition of deubiquitinating enzymes (DUBs) and autophagy synergistically kills breast cancer cells. Oncotarget.

[19] Hanpude P, Bhattacharya S, Dey AK, Maiti TK (2015). Deubiquitinating enzymes in cellular signaling and disease regulation. IUBMB life.

[20] Kobayashi T (2015). Deubiquitinating enzymes regulate Hes1 stability and neuronal differentiation. The FEBS journal.

[21] Wang Y (2015). Deubiquitinating enzymes regulate PARK2-mediated mitophagy. Autophagy.

[22] Gu Z, Shi W (2016). Manipulation of viral infection by deubiquitinating enzymes: new players in host-virus interactions. Future microbiology.

[23] Sisto M, Barca A, Lofrumento DD, Lisi S (2016). Downstream activation of NF-kappaB in the EDA-A1/EDAR signalling in Sjogren’s syndrome and its regulation by the ubiquitin-editing enzyme A20. Clinical and experimental immunology.

[24] Schlicher L (2016). SPATA2 promotes CYLD activity and regulates TNF-induced NF-kappaB signaling and cell death. EMBO reports.

[25] Fan Y (2014). USP21 negatively regulates antiviral response by acting as a RIG-I deubiquitinase. The Journal of experimental medicine.

[26] Pauli EK (2014). The ubiquitin-specific protease USP15 promotes RIG-I-mediated antiviral signaling by deubiquitylating TRIM25. Science signaling.

[27] Pringle LM (2012). Atypical mechanism of NF-kappaB activation by TRE17/ubiquitin-specific protease 6 (USP6) oncogene and its requirement in tumorigenesis. Oncogene.

[28] Wang L (2013). USP4 positively regulates RIG-I-mediated antiviral response through deubiquitination and stabilization of RIG-I. Journal of virology.

[29] Lin R (2017). USP4 interacts and positively regulates IRF8 function via K48-linked deubiquitination in regulatory T cells. FEBS letters.

[30] Kuo RL, Kao LT, Lin SJ, Wang RY, Shih SR (2013). MDA5 plays a crucial role in enterovirus 71 RNA-mediated IRF3 activation. PloS one.

[31] Dickson KM, Bhakar AL, Barker PA (2004). TRAF6-dependent NF-kB transcriptional activity during mouse development. Developmental dynamics: an official publication of the American Association of Anatomists.

[32] Clague MJ, Coulson JM, Urbe S (2012). Cellular functions of the DUBs. Journal of cell science.

[33] Engel E (2014). Identifying USPs regulating immune signals in Drosophila: USP2 deubiquitinates Imd and promotes its degradation by interacting with the proteasome. Cell communication and signaling: CCS.

[34] Cui J (2014). USP3 inhibits type I interferon signaling by deubiquitinating RIG-I-like receptors. Cell research.

[35] Gu Z, Shi W, Zhang L, Hu Z, Xu C (2017). USP19 suppresses cellular type I interferon signaling by targeting TRAF3 for deubiquitination. Future microbiology.

[36] Gurtler C, Bowie AG (2013). Innate immune detection of microbial nucleic acids. Trends in microbiology.

[37] Yoshida R (2008). TRAF6 and MEKK1 play a pivotal role in the RIG-I-like helicase antiviral pathway. The Journal of biological chemistry.

[38] Shi Z (2015). Structural Insights into mitochondrial antiviral signaling protein (MAVS)-tumor necrosis factor receptor-associated factor 6 (TRAF6) signaling. The Journal of biological chemistry.

[39] Lee NR, Kim HI, Choi MS, Yi CM, Inn KS (2015). Regulation of MDA5-MAVS Antiviral Signaling Axis by TRIM25 through TRAF6-Mediated NF-kappaB Activation. Molecules and cells.

[40] Zeng, K.W. *et al*. Natural small molecule FMHM inhibits lipopolysaccharide-induced inflammatory response by promoting TRAF6 degradation via K48-linked polyubiquitination. *Scientific reports* **5**, 14715 (2015). 10.1038/srep14715 PMC4589686 26423026

[41] Zhou L, Ma Q, Shi H, Huo K (2010). NUMBL interacts with TRAF6 and promotes the degradation of TRAF6.. Biochemical and biophysical research communications.

[42] Li, Z. *et al*. USP4 inhibits p53 and NF-kappaB through deubiquitinating and stabilizing HDAC2. *Oncogene* **35**, 2902–2912 (2016). 10.1038/onc.2015.349 PMC4895393 26411366

[43] Mehic M, de Sa VK, Hebestreit S, Heldin CH, Heldin P (2017). The deubiquitinating enzymes USP4 and USP17 target hyaluronan synthase 2 and differentially affect its function. Oncogenesis.

[44] Xiao N (2012). Ubiquitin-specific protease 4 (USP4) targets TRAF2 and TRAF6 for deubiquitination and inhibits TNFalpha-induced cancer cell migration. The Biochemical journal.

[45] Iha H (2008). Inflammatory cardiac valvulitis in TAX1BP1-deficient mice through selective NF-kappaB activation. The EMBO journal.

[46] Karin M, Greten FR (2005). NF-kappaB: linking inflammation and immunity to cancer development and progression. Nature reviews. Immunology.

[47] Hotter D (2017). Primate lentiviruses use at least three alternative strategies to suppress NF-kappaB-mediated immune activation. PLoS pathogens.

[48] Luo Z (2017). HRS plays an important role for TLR7 signaling to orchestrate inflammation and innate immunity upon EV71 infection. PLoS pathogens.

[49] Fan YH (2011). USP4 targets TAK1 to downregulate TNFalpha-induced NF-kappaB activation. Cell death and differentiation.

