# Author Correction: USP4 positively regulates RLR-induced NF-κB activation by targeting TRAF6 for K48-linked deubiquitination and inhibits enterovirus 71 replication

**DOI:** 10.1038/s41598-023-38726-1

**Published:** 2023-07-19

**Authors:** Chao Xu, Yang Peng, Qin Zhang, Xiao-Peng Xu, Xiang-Min Kong, Wei-Feng Shi

**Affiliations:** Department of Laboratory Medicine, The Third Affiliated Hospital of Soochow University, Changzhou, 213003 Jiangsu People’s Republic of China

Correction to: *Scientific Reports* 10.1038/s41598-018-31734-6, published online 07 September 2018

This Article contains an error.

As a result of an error during the figure assembly, in Figure 6B, the panels *Flag* and *GAPDH* of the “Input” are overlapping with the *Flag* and *GAPDH* panels of the “Input” in Figure 6C.

The corrected Figure 6 and its accompanying legend appears below as Figure 1.

**Figure 1:**
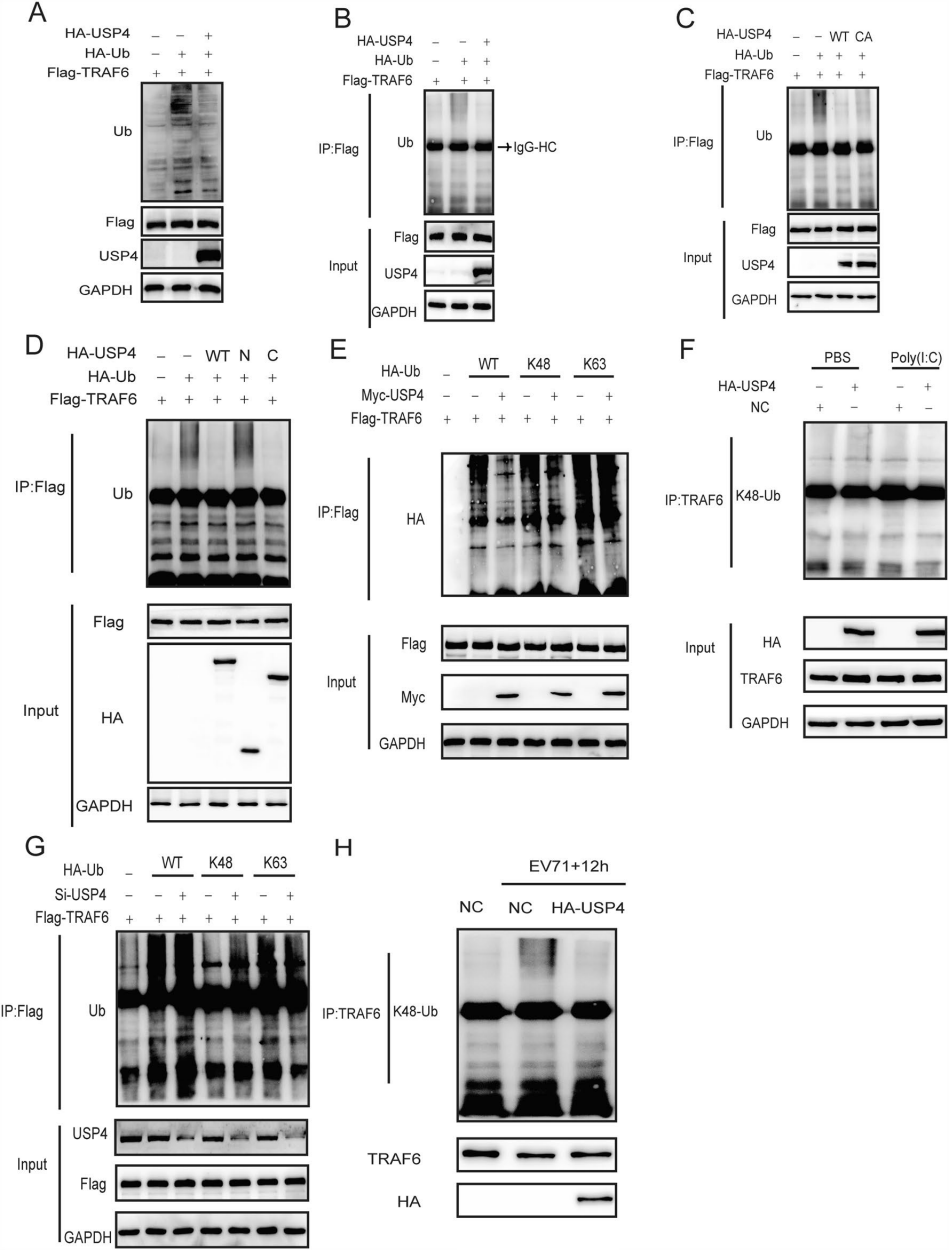
USP4 removes K48-linked polyubiquitination from TRAF6. (**A**) Western blot analysis of HEK293T cells transfected with Flag-TRAF6, HA-USP4, and HA-Ub for ubiquitination levels; GAPDH was used as a loading control. (**B,C**) HEK293T cell lysates transfected with Flag-TRAF6, HA-Ub, together with either wild-type HA-USP4 (WT) or mutated HA-USP4 (CA) were collected and immunoprecipitated with anti-Flag agarose beads. The eluted immunocomplexes was then subjected to SDS-PAGE analysis with anti-ubiquitin antibody. (**D**) Lysates from HEK293T cells transiently expressing Flag-tagged TRAF6 and HA-tagged ubiquitin, together with either HA-tagged USP4 or its truncated constructs (N or C) were immunoprecipitated with anti-Flag agarose beads; the eluted protein complexes was subjected to western blot analysis with anti-ubiquitin antibody. (**E**) HEK293T cells transfected with Flag-TRAF6, Myc-USP4, together with either WT HA-Ub or its mutants (K48 or K63) were harvested and subjected to immunoprecipitation with anti-Flag agarose beads followed by SDS-PAGE analysis with HA antibody. (**F**) Lysates from HEK293T cells transfected with HA-USP4 or a control vector followed by treatment with poly (I:C) (HMW; 30 μg/ml) for 8 h were subjected to immunoprecipitation with an anti-TRAF6 antibody. This was followed by western blot analysis of eluted immunocomplexes with K48 linkage-specific ubiquitin antibodies. (**G**) HEK293T cells transfected with Flag-TRAF6, control siRNA or USP4-specific siRNA, together with either WT HA-Ub or its mutants (K48 or K63) were harvested and subjected to immunoprecipitation with anti-Flag agarose beads followed by SDS-PAGE analysis with anti-ubiquitin antibody. (**H**) Western blot analysis of RD cells transfected with control plasmid or HA-USP4 plasmid for 48 h, followed by treatment with EV71 (MOI = 0.5) for 12 h. The cell lysates were subjected to immunoprecipitation with anti-TRAF6 antibody, followed by western blot analysis of eluted immunocomplexes with K48 linkage-specific ubiquitin antibodies.

