# Supplementary material for: USP4 positively regulates RLR-induced NF-κB activation by targeting TRAF6 for K48-linked deubiquitination and inhibits enterovirus 71 replication
Source: Sci Rep. 2018 Sep 7;8:13418. doi: 10.1038/s41598-018-31734-6 (PMC6128947; doi:10.1038/s41598-018-31734-6)

**USP4 positively regulates RLR-induced NF-κB activation by targeting TRAF6 for K48-linked deubiquitination and inhibits enterovirus 71 replication**

Chao Xu^1^, Yang Peng^1^, Qin Zhang^1^, Xiao-Peng Xu^1^, Xiang-Min Kong^1^,Wei-Feng Shi^1*^

**Figure legend**

**Figure S2. USP54 is not involved in the cellular antiviral immune response to EV71 infection.** (A) phase contrast microscopy analyses of RD cells transfected with empty vector or an expression vector of USP54 ,and then infected with EV71 at an MOI of 0.5 at the indicated time.Original magnification,×20. (B) cell apoptosis assay of EV71-infected RD cells transfected with empty vector or an expression vector of USP54 ,and then infected with EV71 at an MOI of 0.5 at the indicated time. The apoptosis assay was conducted using annexin V/FITC and PI double staining and ﬂow cytometry. Q3, Q4, and Q2 represent normal cells, early apoptotic cells and late apoptotic/necrotic cells, respectively. (C) Viral titer measurement of EV71 propagating in RD cells transiently transfected with control vector or HA-USP54 plasmids.


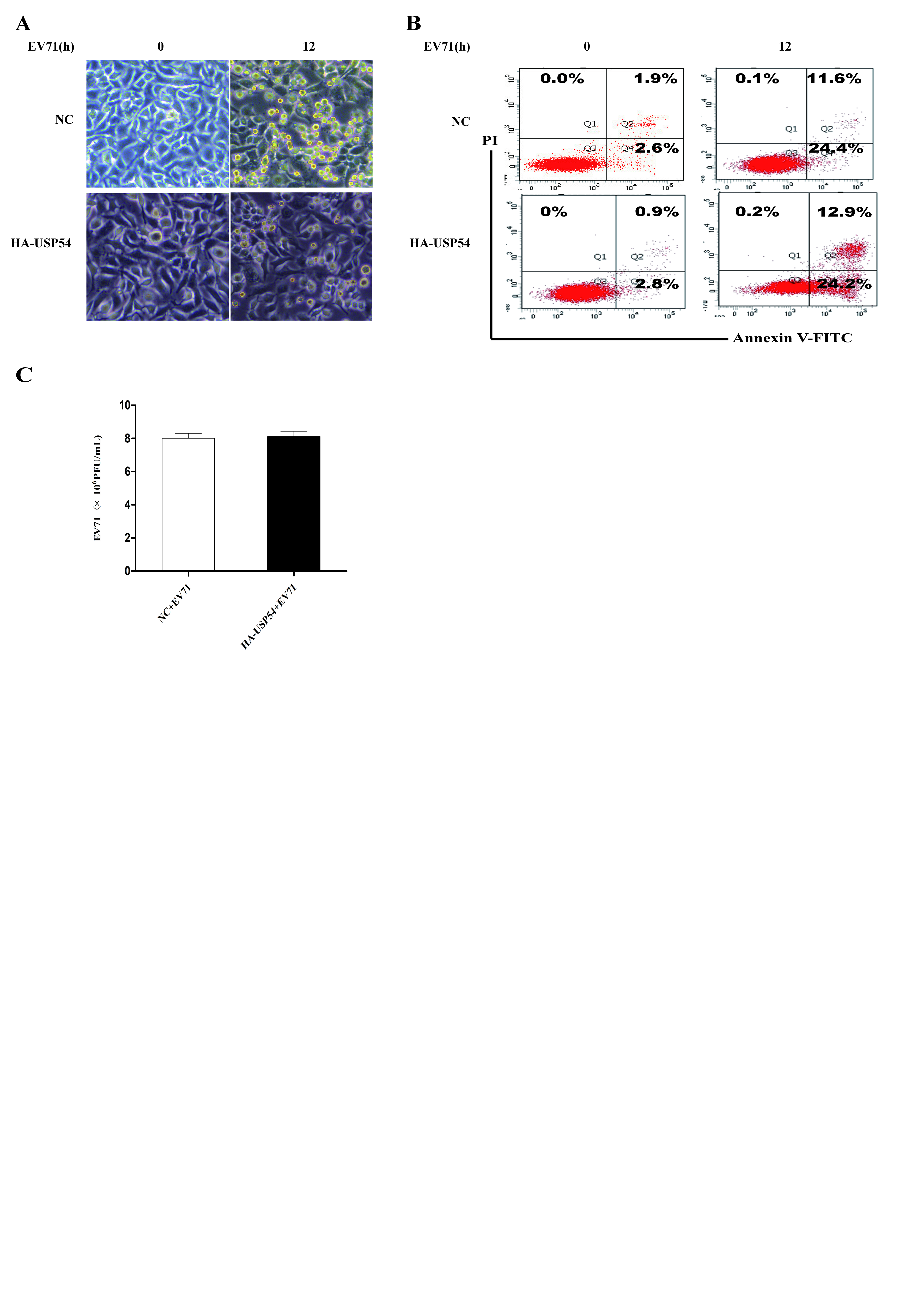

Supplement: Supplementary file 1 — Supplementary Information [file 41598_2018_31734_MOESM1_ESM.docx]
